# High heterogeneity in *Plasmodium falciparum *risk illustrates the need for detailed mapping to guide resource allocation: a new malaria risk map of the Lao People's Democratic Republic

**DOI:** 10.1186/1475-2875-9-59

**Published:** 2010-02-24

**Authors:** Pernille Jorgensen, Simone Nambanya, Deyer Gopinath, Bouasy Hongvanthong, Kongxay Luangphengsouk, David Bell, Samlane Phompida, Rattanaxay Phetsouvanh

**Affiliations:** 1 Center for Malariology, Parasitology and Entomology, Ministry of Health, Ban Chanthabouly, Vientiane, Lao People's Democratic Republic; 2 World Health Organization, Saphanthong Road, Ban Saphanthongtai, Vientiane, Lao People's Democratic Republic; 3 Foundation for Innovative New Diagnostics, Avenue de Budé, Geneva, Switzerland; 4 The Global Fund to fight AIDS, Tuberculosis and Malaria, Principal Recipient Office, Ministry of Health, Simeuang Road, Vientiane, Lao People's Democratic Republic

## Abstract

**Background:**

Accurate information on the geographical distribution of malaria is important for efficient resource allocation. The Lao People's Democratic Republic has experienced a major decline in malaria morbidity and mortality in the past decade. However, efforts to respond effectively to these changes have been impeded by lack of detailed data on malaria distribution. In 2008, a countrywide survey on *Plasmodium falciparum *diagnosed in health centres and villages was initiated to develop a detailed *P. falciparum *risk map with the aim to identify priority areas for malaria control, estimate population at risk, and guide resource allocation in the Lao People's Democratic Republic.

**Methods:**

*P. falciparum *incidence data were collected from point-referenced villages and health centres for the period 2006-2008 during a country-wide survey between December 2008 and January 2009. Using the highest recorded annual rate, continuous surfaces of *P. falciparum *incidence were produced by the inverse distance weighted interpolation technique.

**Results:**

Incidence rates were obtained from 3,876 villages and 685 health centres. The risk map shows that *P. falciparum *is highly heterogeneous in the northern and central regions of the country with large areas of no transmission. In the southern part, transmission is pervasive and the risk of *P. falciparum *is high. It was estimated that 3.4 million people (60% of the population) live at risk of malaria.

**Conclusions:**

This paper presents the first comprehensive malaria risk map of the Lao People's Democratic Republic based entirely on empirical data. The estimated population at risk is substantially lower than previous estimates, reflecting the presence of vast areas with focal or no malaria transmission as identified in this study. These findings provide important guidance for malaria control interventions in the Lao People's Democratic Republic, and underline the need for detailed data on malaria to accurately predict risk in countries with heterogeneous transmission.

## Background

Malaria risk mapping is increasingly being utilized by national malaria programmes as a tool for planning and guiding malaria control strategies [1-6]. Detailed knowledge of the geographical extent of malaria and intensity of transmission can guide decisions on appropriate and cost-effective control strategies, allow monitoring of the impact of interventions, identify areas with epidemic potential, and provide more accurate estimates of the burden of disease and population at risk.

In the Lao People's Democratic Republic (Lao PDR), malaria is considered endemic throughout the country, but intensity of transmission is known to vary between different ecological zones; from very low transmission in the plains along the Mekong river and in areas of high altitude, to intense transmission in remote, hilly and forested areas (Laura Watson: Malaria in Lao PDR - a Review, unpublished report). *Plasmodium falciparum *is the predominant species accounting for 95% of all recorded malaria cases [7]. Transmission of malaria is perennial, but with large seasonal and regional variations. Peak transmission occurs between May and October, coinciding with the hot and rainy season [7].

Malaria has long been a leading cause of mortality and morbidity in the country [7,8]. However, significant reductions have been reported after recent investments in malaria control, in particular the large-scale introduction of artemisinin-based combination therapy (ACT) and insecticide-treated nets (ITNs), in conjunction with socio-economic and environmental changes. In 2008, only 11 deaths among 18,743 confirmed malaria cases were reported (population 6 million), compared with 600 deaths and 70,000 confirmed cases in 1997 (routine malaria surveillance reports, Center for Malariology, Parasitology and Entomology (CMPE), unpublished data).

These recent changes have important implications for the planning and design of malaria control interventions, including resource allocation. Nevertheless, up till now efforts to respond have been impeded by a lack of reliable data and maps which accurately reflect the contemporary distribution of malaria in the country. Although, global models to predict malaria transmission areas have included Lao PDR [9-12], these have been based on either malaria vector distribution, or a limited number of prevalence surveys in combination with surveillance and climate data, resulting in crude estimates. While giving potentially useful data on global malaria distribution, these broad predictive models are of limited value for guiding resource allocation within national malaria programmes.

Between 2005 and 2008, the national malaria programme introduced a new strategy to improve case management at community level, involving training of 12,404 village health volunteers (VHVs) in 6,202 villages in the use of *P. falciparum-*specific malaria rapid diagnostic tests (Paracheck-Pf^®^, Orchid Biomedical Systems, Goa, India), to guide administration of ACT. The VHVs constitute the most peripheral level of the public health care system in Lao PDR. Volunteers are selected by villagers and the village committee to provide primary health care services, including diagnosis and management of respiratory diseases, diarrhoea, and uncomplicated malaria as well as performing health education, assist in vaccinations campaigns and insecticide treatment of bed-nets, and report morbidity and mortality data to health centre or district health office. The responsibilities of VHVs may vary between locations. It is generally required that VHVs can read and write Lao.

Through the large-scale introduction of RDTs and ACT, information on malaria incidence has now become available at village level, providing a unique opportunity to obtain a more complete picture of the areas and populations affected by malaria throughout the country.

In late 2008, CMPE therefore conducted a countrywide survey of *P. falciparum *cases diagnosed by malaria RDTs in health centres and villages between 2006 and 2008. Using point referenced incidence data, the survey aimed to develop a detailed map of *P. falciparum *risk and identify priority areas for malaria control and resource allocation. This paper reports the results of this effort.

## Methods

### Malaria surveillance in the Lao PDR

The Lao PDR national surveillance system for malaria is based on passive case reporting by provincial and district hospitals, provincial malaria stations, health centres, and VHVs. Malaria diagnosis in malaria stations and hospitals is based on both microscopy and RDTs, while health centres and VHVs predominantly use RDTs. All health facilities and VHVs are requested to report suspected and confirmed malaria cases along with demographic information, number of persons tested by microscopy or RDT, clinical manifestations, and treatment provided. However, reporting is poor in some areas, particularly from VHVs, making aggregated data from district level unreliable. Further data losses are known to occur through incomplete reporting from districts to CMPE.

### Survey on *P. falciparum*

Between December 2008 and January 2009, a nationwide survey was conducted to collect data on confirmed *P. falciparum *cases recorded in all villages and health centres with RDTs or microscopy in the three-year period 2006-2008. A standard data collection form was prepared for each district with the following information: Name of all health centres and villages with RDTs or microscopy in the district, unique administrative code for each village and health centre, date when RDTs were first received, population (village or health centre catchment population), number of persons tested for malaria and number of confirmed *P. falciparum *cases by month between 2006 and 2008. Village-level data was collected from VHV records by district malaria personnel and health centre staff following a two-day training programme. Health centre staff also collated this data from their own facility. In total, 135 district and 755 health centre staff participated in the data collection, covering all districts in the 17 provinces. Five districts in Vientiane Capital which are considered malaria-free were excluded.

Data were entered in a database (*Microsoft Access *2003) containing the geographical coordinates of all villages and health centres, acquired from the National Geographical Department (2005 census). Data on malaria diagnosed in district hospitals between 2006 and 2008 were collected by CMPE in a separate survey in January 2009.

### Statistical and spatial analysis

For each village and health centre an overall incidence of *P. falciparum *per 1,000 population was estimated using the highest recorded annual rate between 2006 and 2008. Continuous surfaces of *P. falciparum *incidence were produced by averaging the incidence rate for all villages and health centres using the inverse distance weighted (IDW) interpolation technique with a fixed search radius of 10 km. A power of 2 was chosen to place more weight on nearest points. The analysis was performed with ArcView 3.3 and Spatial Analyst 1.0, Environmental Systems Research Institute (ESRI).

To quantify malaria risk, *P. falciparum *incidence zones were created using the following classification: 1) 0 case per 1,000 per annum; 2) >0-1 case per 1,000 per annum; 3) >1-10 cases per 1,000 per annum; 4) >10-100 cases per 1,000 per annum; 5) >100-1,000 cases per 1,000 per annum; and 6) unknown (villages where data was not collected and an estimated value could not be produced because the village fell outside the 10 km prediction area). The lower incidence level of less than one case per 1,000 persons has recently been proposed as an operational index to help determine whether an area can move from pre-elimination to elimination phase [13].

The population at risk was estimated by adding the population of villages falling within each of the risk zones according to above classifications. Areas located >10 km from a village centre were considered unpopulated.

All statistical analyses were performed with Stata 10 (StataCorp, Colleague Station, TX, USA).

## Results

### Characteristics of villages and health centres surveyed

Between 2006 and 2008, 5,239 of 10,585 villages located in malaria endemic districts had received RDTs and had at least one VHV trained in malaria diagnosis and treatment. Due to budget constraints, however, data collection was restricted to 4,403 villages. Villages excluded from the survey were mainly those which had not received a malaria register book or where there was no evidence of VHVs actively seeing and testing patients. Several villages were also excluded due to difficulties with access; these were mainly located in the north-eastern part of the country.

A total of 706 (93.1%) of the 758 health centres in endemic districts had RDTs or microscopy. Of these, 702 were included in the survey. The four health centres excluded received RDTs in October 2008 and had not tested patients prior to the survey.

Excluded villages were smaller (354 inhabitants on average; 95% CI: 338-369) than those included (447 inhabitants on average; 95% CI: 439-455), t = 8.94, p < 0.001. Distance (km) to nearest health centre (measured in Euclidean distance) was also longer for excluded villages compared to included villages (6.1 km; 95% CI: 5.7-6.4 versus 5.2 km; 95% CI: 5.3-5.5, t = 5.19, p < 0.001). The proportion of surveyed villages among all villages with RDTs were similar across the central and southern part of the country, 92.5% and 93.2% respectively, while in the northern provinces 65% of villages were included (χ^2 ^= 675.8, p < 0.001).

### Data availability

All survey forms were returned to CMPE. Of the 4,403 villages and 702 health centres included in the survey, 3,876 (88%) and 685 (98%) respectively had tested patients for malaria between 2006 and 2008, providing information on *P. falciparum *incidence for a total of 4,561 geographical locations. About half of the villages and health centres had records on patients tested for malaria in five months or more for the year included in the analysis (Table 1).

**Table 1:** Number of months for which data records on patients tested for malaria was available in health centres and villages for the year with the highest recorded incidence rate of *P. falciparum *between 2006 and 2008.

Number of months	Number of health centres and villages
1	502 (11.0)
2	477 (10.5)
3	519 (11.4)
4	455 (9.9)
5	482 (10.6)
6	486 (10.7)
7	494 (10.8)
8	323 (7.1)
9	272 (5.9)
10	176 (3.9)
11	175 (3.8)
12	200 (4.4)

Total	4,561

### Incidence of *P. falciparum *by province, 2008

The total number of *P. falciparum *cases recorded in Lao PDR in 2008, according to routine data recorded at malaria stations, provincial and district hospitals, and data collected in this survey, were 25,388. Of these 19,418 (76%) were diagnosed by VHVs and health centres. *P. falciparum *incidence rates reported by VHVs and health centres varied substantially between and within provinces, implying skewed distributions of incidence rates with a relatively few high-transmission villages increasing the overall incidence (Table 2).

**Table 2:** Overall and median incidence of confirmed *P. falciparum *per 1,000 population* by province (ranked from highest to lowest) in the Lao People's Democratic Republic, 2008.

Province (number of villages and health centres surveyed)	Overall incidence of *P. falciparum *per 1,000 population	Median incidence of *P. falciparum *per 1,000 population	Min-max incidence of *P. falciparum *per 1,000 (standard deviation)
Sekong (143)	32.7	23.5	0-783.0 (109.0)
Attapeu (172)	24.7	14.3	0-208.0 (28.6)
Savannakhet (495)	11.5	4.6	0-200.5 (27.7)
Saravane (391)	9.6	4.7	0-362.2 (50.4)
Champasack (540)	3.3	0	0-98.2 (9.5)
Khammouane (173)	2.9	0	0-103.0 (15.8)
Phongsaly (94)	2	0	0-100.0 (15.8)
Luangprabang (608)	0.9	0	0-62.5 (5.5)
Bokeo (82)	0.7	0	0-20.7 (2.4)
Oudomxay (128)	0.7	0	0-38.1 (5.6)
Luangnamtha (72)	0.4	0	0-8.8 (1.3)
Xaignabury (153)	0.1	0	0-18.7 (1.6)
Xiengkhuang (285)	0.1	0	0-11.6 (0.8)
Bolikhamxay (228)	0.1	0	0-27.1 (1.8)
Vientiane (151)	0.1	0	0-7.2 (1.1)
Huaphan (334)	0.1	0	0-32.0 (1.8)
Vientiane Capital (57)	0.01	0	0-6.5 (1.0)

* Cases diagnosed by rapid diagnostic tests in villages and health centres (n = 4,106) in 2008 as recorded during a country wide survey conducted between December 2008 and January 2009. Malaria cases diagnosed by provincial and district hospitals, and malaria stations are excluded.

### Geographical distribution and risk of *P. falciparum*

**Figure 1:**
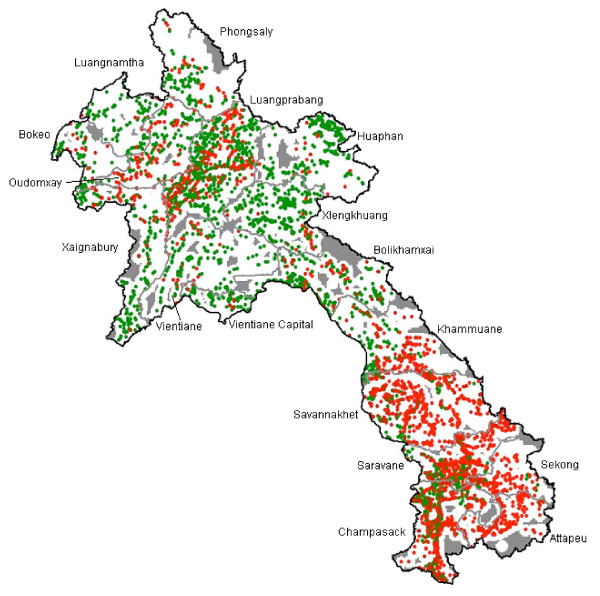
**Distribution of *P. falciparum *cases diagnosed by health centres and villages included in the survey**. Red dots: Villages or health centres reporting at least one confirmed case of *P. falciparum *between 2006 and 2008, green dots: Villages or health centres which did not confirm any case of *P. falciparum *between 2006 and 2008. Grey areas signify unpopulated areas (areas >10 km from a village centre).

**Figure 2:**
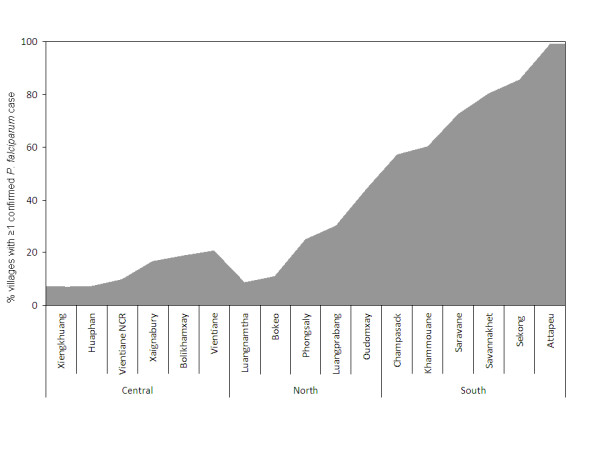
**Proportion (%) of villages recording at least 1 confirmed case of *P. falciparum *among all villages with RDTs between 2006 and 2008 by province and region (number of villages = 3,876)**.

The majority of villages and a large proportion of health centres did not confirm any *P. falciparum *cases between 2006 and 2008 (Table 3). The geographical distribution of confirmed *P. falciparum *cases in the period 2006-2008 is summarized in Figure 1. A significantly higher proportion of villages in the south (72.3%, n = 1,937) recorded *P. falciparum *cases compared with locations in the northern (28.3%, n = 962) and central (12.7%, n = 977) part of the country (χ^2 ^= 1.2e+03, p < 0.001) (Figure 2). Annual recorded incidence rates in villages varied substantially from 0 to 815 per 1,000 per year, with the highest rates being in the southern provinces of the country. At health centre level, *P. falciparum *incidence ranged from 0 to 167 confirmed cases per 1,000 population per year.

**Table 3:** Highest recorded *P. falciparum *incidence rate for any year between 2006 and 2008 by villages and health centres with RDTs (n = 4,561)

Annual incidence rate of *P. falciparum *per 1,000 population	Number of villages (% of total)	Number of health centres (% of total)
0	2,079 (53.6)	315 (46.0)
>0-1	23 (0.6)	138 (20.2)
>1-10	842 (21.7)	156 (22.8)
>10-100	778 (20.1)	70 (10.2)
>100-1,000	154 (4.0)	6 (0.9)

Total	3,876	685

**Figure 3:**
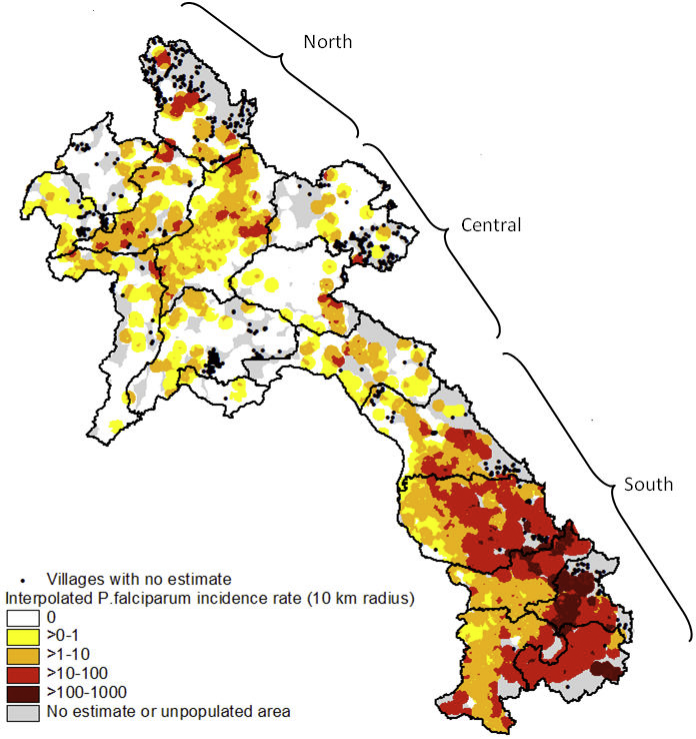
**Interpolated highest annual incidence rates of *P. falciparum *based on data from 3,876 villages and 685 health centres (2006-2008)**. Black dots show villages not included in the survey for which estimates on incidence were not available. Northern provinces: Phongsaly, Luangnamtha, Luangprabang, Bokeo, and Oudomxay; Central provinces: Huaphan, Xiengkhuang, Vientiane, Vientiane Capital, Xaignabury, and Bolikhamxay; and Southern provinces: Khammuane, Savannakhet, Saravane, Sekong, Champasack, and Attapeu.

The interpolated (highest) annual incidence rate of *P. falciparum *recorded in the three-year period is shown in Figure 3. Based on this risk map, three geographical zones could roughly be delineated: low risk in the central part of the country, where *P. falciparum *was only reported in few locations, generally with very low incidence rates; intermediate risk in the northern provinces, where *P. falciparum *also exhibited geographical focality but where transmission appeared more widespread in general and incidence rates were higher; high risk in the southern zone, where *P. falciparum *transmission was pervasive with very high incidence rates particularly in villages along the border to Vietnam.

**Figure 4:**
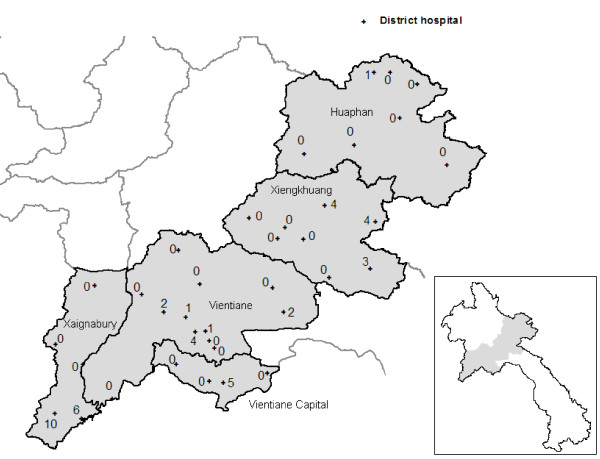
**Map showing total number of confirmed *P. falciparum *cases recorded by district hospitals in 2008 in five central provinces of Lao PDR**.

Data on malaria collected from district hospitals showed a similar pattern. In 2008, the total number of confirmed *P. falciparum *cases recorded were 4,083 in hospitals in the southern part of the country (six provinces, population 2.5 million), 481 in the northern part (five provinces, population 1.2 million), and 49 in the central zone (six provinces, population 2.3 million (Figure 4)).

### Population at risk

**Figure 5:**
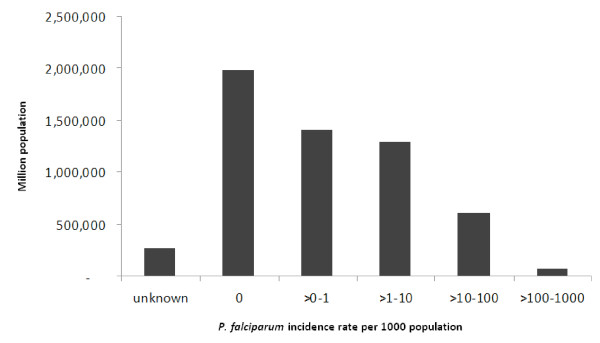
**Estimated population at risk of *P. falciparum *in Lao PDR by interpolated incidence rate group (population data from 2005 census)**.

A total of two million people (35% of the population) were living in areas where the predicted annual risk of *P. falciparum *was zero (Figure 5). Adding villages with an incidence below one per 1,000, it was estimated that 3.4 million people (60% of the population) currently live in zones which fulfil the criteria for elimination stage.

## Discussion

This paper presents the first detailed map of malaria in the Lao PDR providing novel insights into the contemporary geographical distribution and risk of *P. falciparum *in the country, underlining the highly uneven distribution with which malaria transmission may occur. The results, based on over 4,500 well-distributed point-referenced annual incidence data from villages and health centres, indicate high spatial heterogeneity in the central and northern regions of the country with large areas where transmission is not recorded. In the south, more uniform high risk is present but also pockets of very high and low risk. These findings are essentially consistent with established classification of overall malaria endemicity in the Lao PDR, but the high variability and vast areas with no or unstable risk has hitherto not been described.

The level and extent of malaria transmission results from interaction between several factors, including the parasite, *Anopheline *vectors, human host behaviour, malaria control interventions, and climate [14]. Socio-economic status has also been shown to play a role [15]. While this study did not investigate risk factors associated with malaria, some broad determinants discussed below can partly explain the patterns observed.

The main malaria vectors in the Lao PDR are members of the *Anopheles dirus *and the *Anopheles minimus *complexes [16]. While the former has been identified predominantly in the south [17,18], the latter occurs throughout the country [7]. The presence of *An. dirus *in southern Lao PDR coincides with high risk areas identified in this study. This mosquito is highly anthropophilic and an efficient malaria vector even at low densities [18-20]. Furthermore, the early biting and exophilic behaviour of the mosquito[19-22] can help explain the continued high incidence of *P. falciparum *in the south-eastern part of the country despite a reportedly high ITN coverage. The higher endemicity in the south may also be a result of the presence of both *An. dirus *and *An. minimus*, the latter also being active during the drier part of season, providing ideal conditions for sustained perennial transmission [17,20].

Conversely, large-scale distribution of ITNs in recent years may have contributed significantly to reduction of *An. minimus *populations and thus malaria in the northern and central part. It has been reported that the vector disappeared from many areas in Asia during former large-scale indoor residual spraying (IRS) campaigns although behavioural shifts to outdoor feeding and zoophilic preferences were observed and transmission was never fully interrupted [20,23]. The highly seasonal nature of malaria transmission in the northern and central part of the country may also render these regions more susceptible to malaria control measures; in several locations in the mountainous central and northern provinces the monthly average temperatures drop below 19°C, the minimum temperature required for *P. falciparum *sporogony [24], for up to three consecutive months.

This study estimated that two million (35% of the population) live in areas where the annual incidence of *P. falciparum *was predicted to be zero. This figure is substantially higher than previous published estimates which have suggested that only a very small fraction of the population live in areas of no or very low risk [10-12,25]. The discrepancies between the data presented here and previous estimates is likely due to two main factors: 1) maps based on few input points in a highly heterogeneous area may overestimate the population at risk, particularly if based on studies designed for other reasons and targeting known high-transmission sites; and 2) the use of contemporary data in this study compared to older prevalence studies which may not accurately reflect recent changes in the epidemiology and transmission intensity of malaria in the Lao PDR.

Basing a malaria risk map on routinely recorded data as the one presented here also has a number of limitations. Firstly, our data relied on *P. falciparum *cases diagnosed by RDTs at health centres and villages. While, RDT-based diagnosis can be very accurate [26], quality of training, intensity of transmission, and storage conditions among others can greatly affect performance of the tests [27-30]. Accuracy and completeness of patient recording by health workers will also influence results, either over- or underestimating incidence. In the Lao PDR, RDTs are routinely lot-tested prior to and during use in the field, making poor performance unlikely.

Secondly, data on *P. falciparum *was not obtained from a number of the most remote villages in the country, predominantly in the northern region. Although this may have resulted in an underestimation of the population at risk, the excluded areas are relatively sparsely populated, and the major difference between the results presented here and previously published estimates are more likely due to a lower estimate for the central region, which was well covered in this study. The exclusion of district and provincial hospitals from the risk map could have the same result, but was considered appropriate as these health facilities are unlikely to represent the geographical origin of malaria cases.

Thirdly, as the risk map was based on the highest incidence recorded in a three year period it will overestimate the current incidence of *P. falciparum *in some areas, particularly in the central part of the country where a number of districts did not report any cases in 2008 through the routine surveillance system. Conversely, the reliance on public sector data exclusively and the inclusion of villages and health centres with only one month of malaria data records will underestimate *P. falciparum *risk. However, low malaria testing rates observed in some villages may partly be compensated for by villagers seeking treatment in neighbouring villages as suggested by anecdotal evidence.

Lastly, the IDW method assumes positive autocorrelation, i.e., that locations closer to each other are more similar than locations further away. While investigation of spatial autocorrelation was beyond the scope of this study, the existence of spatial clustering of malaria incidence within and between villages in other settings has previously been described [2,31-33]. Furthermore, gradually diminishing incidence of malaria by increasing distance from defined *Anopheles *breeding sites over 10 km has been observed [6]. The presence of such risk gradients in Lao PDR is likely as vector density decreases with distance to deep forest [22]. Since the vast majority (80%) of villages in the Lao PDR are within 2.5 km of another village, which is largely the dispersal range of both *An. dirus *and *An. minimus *[16,22], the use of the IDW method to interpolate malaria incidence rates appears justified.

Detailed knowledge of the spatial distribution and burden of malaria is essential not only for planning and prioritizing resources, but also because uniform application of malaria control strategies may lead to an overall decrease in incidence but result in sustained transmission in foci with higher transmission potential [34]. Although past mapping of malaria incidence at district level has helped to elucidate the burden of disease in a spatial manner, it has not provided a sufficiently accurate picture of *P. falciparum *distribution to inform targeted control and prevention efforts, particularly in areas of focal and heterogeneous transmission. Likewise, other approaches for generating continuous endemicity maps based on a combination of empirical data and models have had limited application at country level because these rely on extrapolation to large areas which may not share the same epidemiology. Such maps may reflect receptivity of an area to malaria, but fail to adequately take into account the effects of control measures and case management to determine actual transmission.

While the risk map presented here has a number of limitations, it has overcome some of the deficiencies of previous maps by quantifying malaria risk at a much smaller scale and by providing a contemporary picture of the distribution of malaria and the population at risk. Furthermore, it has offered a baseline from which to measure the impact of future malaria control interventions, and has showed that a smaller proportion of the population is at risk of malaria than previously assumed, with important implications for resource allocation. The results has also emphasized the need for an increased focus in the south-eastern part of the country which carries the major burden of malaria, but also has the highest poverty rates [35]. Finally, in the long-term, it is unlikely that provision of RDTs, ACT, and malaria case management training for VHVs can be sustained in villages with no reported malaria cases in the last three years. The results of this survey will allow the malaria programme to reallocate its activities in a way that has minimal impact on overall access to malaria treatment and that addresses high transmission foci. Future studies should seek to understand the factors determining the spatial pattern and variation in risk in order to refine choice of interventions.

## Competing interests

The authors declare that they have no competing interests.

## Authors' contributions

DG, BH, RP, PJ, SN, and SP conceived, designed, planned, and coordinated the study. All authors based in Lao PDR participated in the implementation and coordination of training and data collection. KP and PJ supervised and participated in data entry. PJ and KP verified and analyzed the data. All authors contributed to the data interpretation. PJ drafted the first manuscript. DB and DG critically reviewed and revised the first draft of the manuscript. All authors have contributed to and approved the final manuscript
